# Regional bone density patterns of the tibial plateau: implications for finite element analysis

**DOI:** 10.3389/fbioe.2025.1541536

**Published:** 2025-05-22

**Authors:** Pablo Besa, Anselmo Alegría, Nicolás González, Fiorella Biancardi, Catalina Vidal, Pablo Cikutovic, Marcelo E. Andia, Joaquín Mura

**Affiliations:** ^1^ Department of Orthopedic Surgery, School of Medicine, Pontificia Universidad Católica de Chile, Santiago, Chile; ^2^ Radiology Department, School of Medicine, Pontificia Universidad Católica de Chile, Santiago, Chile; ^3^ Biomedical Imaging Center, Radiology Department, School of Medicine, Pontificia Universidad Católica de Chile, Santiago, Chile; ^4^ Department of Mechanical Engineering, Universidad Técnica Federico Santa María, Santiago, Chile

**Keywords:** tibial plateau anatomy, finite element modeling, bone density, tibial fracture, bone architecture

## Abstract

The tibial plateau has different anatomical regions and heterogeneous bone densities. Most finite element simulation (FEM) studies of tibial plateau fracture fail to account these regional variations, which may significantly influence biomechanical behavior. This study aimed to quantify the regional density profile of the tibial plateau using Hounsfield Units (HU) from computed tomography (CT) scans and explore associations between density, age, and sex. We developed a novel measurement protocol to compare HU values of the subchondral bone and cancellous bone in eight different regions of the tibial plateau. Results demonstrated that patient age and female sex were associated with reduced bone density. Subchondral bone and medial bone had significantly higher density than metaphyseal and lateral bone, respectively. This findings could have implications on orthopedic modeling of tibial plateau fractures using FEM. Current FEM should consider distinct regions in tibial plateau to improve accuracy. Conclusion: Tibial plateau heterogenous bone density distribution could contribute to explain the low predictive accuracy in FEM models.

## Introduction

Tibial plateau fractures are among the most common fractures of the knee (Malik et al., 2024). A substantial proportion of these fractures require surgical intervention to achieve good medium to long-term results and expedite patient recovery (McNamara et al., 2015). Despite advances in surgical techniques, there remains a significant number of patients experiencing prolonged work leaves (McNamara et al., 2015), suboptimal mid to long-term outcomes, and inadequate recovery from their fractures (Reátiga Aguilar et al., 2022). Consequently, computational approaches like finite element method (FEM) modeling have emerged as critical tools for studying fracture mechanics and optimizing fixation strategies (Aubert et al., 2021; Zeng et al., 2022).

FEM allows the digital replication of bone for biomechanical simulations. Its primary advantage lies in their ability to test multiple scenarios and countless possibilities. In essence, we can test various fixation solutions within the digital realm of patient-specific models. The precision of the FEM is positively correlated with its complexity (Irarrázaval et al., 2021; Imai et al., 2006), indicating that more accurate predictions result from truer simulations of real bone geometry, mechanical properties, and different tissues interfaces. Current tibial plateau FEM models of the tibial plateau, however, often oversimplify cancellous bone as isotropic and homogeneous (Aubert et al., 2021; Zeng et al., 2022; Gao et al., 2022). This contradicts clinical observations, the subchondral region is considerate denser than the rest of the metaphyseal bone, and the medial plateau sustains higher axial loads, implicating greater density to the lateral plateau (Krause et al., 2018). Surgeons routinely account for these and other regional densities into consideration.

While prior studies have previously explored anisotropic bone properties, approaches rely on laborious, element-by-element density-based FEMs (Chen et al., 2017; Ferre et al., 2022). However, these models are too complex for practical use in patient-specific FEM simulation. Furthermore, no previous studies have assessed the number of discrete density regions in the tibial plateau. Additionally, if there are different densities in subregions of the tibial plateau, no previous study has demonstrated the different regional density of this bone region.

This study aimed to determine the regional density profile of the tibial plateau and explore the association of the regional density profile with age and sex.

## Methods

Institutional review board approval was obtained to conduct this study (IRB 230226001).

We retrospectively analyzed consecutive skeletally mature patients who underwent knee computed tomography (CT) at a tertiary care university hospital. Scans were manly indicated for knee trauma (e.g., knee torsion, ligament injury suspected) and patellofemoral instability. Exclusion criteria included tibial plateau fractures, tumors, infections, or previous knee surgeries (e.g., hardware, tunnels, etc.).

### Imaging protocol

CT images were acquired with a 64-section multidetector CT scanner (Philips Brilliance CT 64-slice), using 64 × 0.6 mm collimation, with a pitch of 1.3. CT was performed with the knee in full extension, approximately total exposure time 10–15 s. Routine multiplanar reconstructions were done in standard sagittal and coronal planes: slice thickness of 0.8 mm and reconstruction increment of 0.4 mm. 768 × 768 square matrix of pixels.

### Tibial density profile

To determine regional tibial densities, we created a standardized measurement protocol. We used Hounsfield Units as a tibial density proxy. HU has shown to correlate with bone density positively and strongly when measured in cancellous bone (Rho et al., 1995). Images were assessed using the Impax Web5000 program (Agfa-Gavaert, Mortsel, Belgium). Contrast was set to bone and windows were set to a 2 × 1 grid, showing axial and coronal views of the tibia.

### Regions of interest (ROI) definition

**FIGURE 1:**
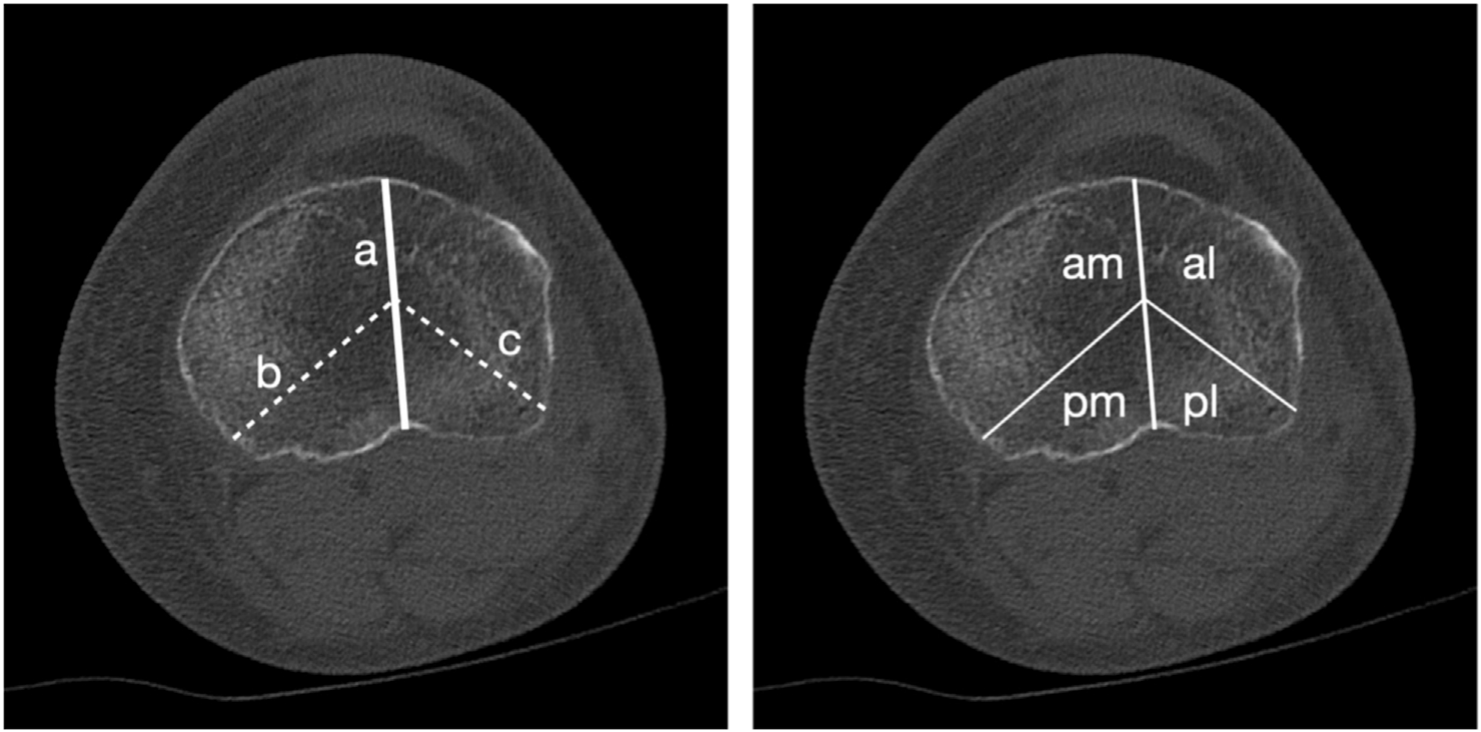
Axial slice of a CT scan of the tibial plateau, just above the tip of the fibula. Left side shows three lines: a, from the tibial tubercle to the posterior cruciate sulcus; b, from the posteromedial cortex to the midpoint of line a; and c, from the region of the fibula in the posterolateral corner to the midpoint of line a. Right side shows the same slice, now marking.

**FIGURE 2:**
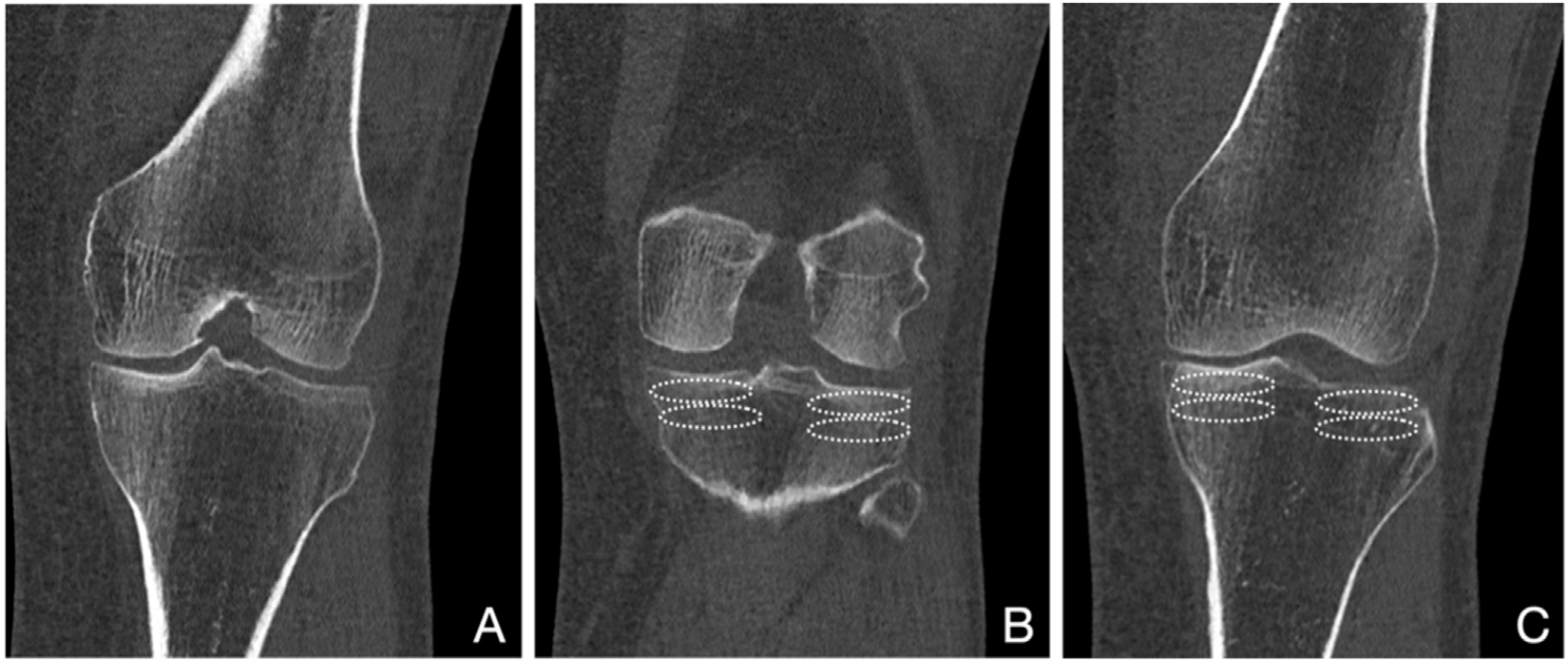
Coronal views of a CT scan of the tibial plateau and distal femur. **(A)** shows the midline cut were the lines a, b and c of Figure 1 meet. **(B)** shows three slices back, with the ellipses to measure HU in the posterolateral and posteromedial subchondral and metaphyseal regions. **(C)** shows three slices forward, with the ellipses to measure HU in the anterolateral and anteromedial subchondral and metaphyseal regions.

The axial cuts were scrolled until just proximal to the fibular apex. As described by Luo et al. (Luo et al., 2010), in this three-column fixation, the tibia was divided into four regions: anteromedial, anterolateral, posteromedial, and posterolateral. These areas, depicted in the axial view (Figure 1) were used to sample the coronal view. Posterior measurements were done four coronal slices behind the depicted midline, while the anterior measurements were done four coronal slices anterior to the midline. To measure the HU of the areas, we drew a 5 mm high and size between the tibial spine and the cortical bone limit diameter ellipse. This was drawn in subchondral metaphysial bone and the next 5 mm ellipse area below (Figure 2) considerating not measure cortical bone.

We drew four regions of interest (ROI) in each coronal slice measured, two medial and two lateral. The most proximal ROIs measured the HU in the first 5 mm (mm) of subchondral bone by drawing a standardized ellipse in this region limited of the cortical bone and tibial spine. Distal to these ROIs, we drew a second medial and a second lateral ellipse, including the next 5 mm (Figure 2), capturing the HU of the metaphyseal (not subchondral) cancellous bone. Each ROI was repeated in adjacent coronal slices, and results for each region were averaged. This produced HU for eight distinct tibial regions: four subchondral and four metaphyseal cancellous.

To validate this *de novo* protocol, repeat measurements were taken in 240 regions by two independent authors. Both reviewers were medical students that were trained for the measurement. The agreement between measurements was obtained using the intraclass correlation coefficient (ICC), a two-way random model characterized by absolute agreement (Hallgren, 2012). Interpretation of ICC was done following the recommendation of Fleiss (Fleiss, 2011), where ICC above 0.74 is considered an excellent agreement.

### Statistical analysis

Statistics were conducted using Stata v17 (StataCorp. 2021. Stata Statistical Software: Release 17. College Station, TX: StataCorp LLC.). We tested normality using the Shapiro-Wilk test. We used paired t-Test to compare mean HU values of metaphyseal bone versus subchondral bone and one-way paired ANOVA test to compare mean HU values for all regions with Bonferroni correction for multiple comparisons. Also, we compare measures with patient sex (Independent t-Test) and age (Spearman correlation coefficient). Normal data is shown as mean and standard deviation (SD); and non-normal data is shown as median and interquartile (IQ) range. Significance was set at 5%.

## Results

We analyzed 102 CT scans of healthy proximal tibias. The cohort comprised 65% female patients (66/102), with a median age of 61 years (interquartile range [IQR]: 35–72 years; range: 15–94 years). Tibial bone density, as measured by HU, followed a normal distribution (Shapiro-Wilk p > 0.05 for all regions).

### Subcondral vs. metaphyseal bone density

Subchondral bone (318.9 ± 131.2 HU) had a significantly higher density than the metaphyseal bone (166.9 ± 71.1 HU) in all the compared regions (p < 0.01) (Table 1)

**TABLE 1:** Mean, standard deviation (SD) and 95% confidence interval (CI) of the density Hounsfield Units of the 4 subchondral and metaphyseal bone regions.

Region	Metaphyseal			Subchondral			paired t-test
	Mean (HU)	SD	95%CI	Mean (HU)	SD	95%CI	p-value
Anterolateral	146.5	61.8	12.0	264.9	111.5	21.6	<0.001
Posteromedial	167.5	82.6	16.0	341.8	152.1	29.5	<0.001
Posterolateral	172.6	70.6	13.7	311.1	113.3	22.0	<0.001
Anteromedial	180.8	82.9	16.1	357.8	148.7	28.8	<0.001

**FIGURE 3:**
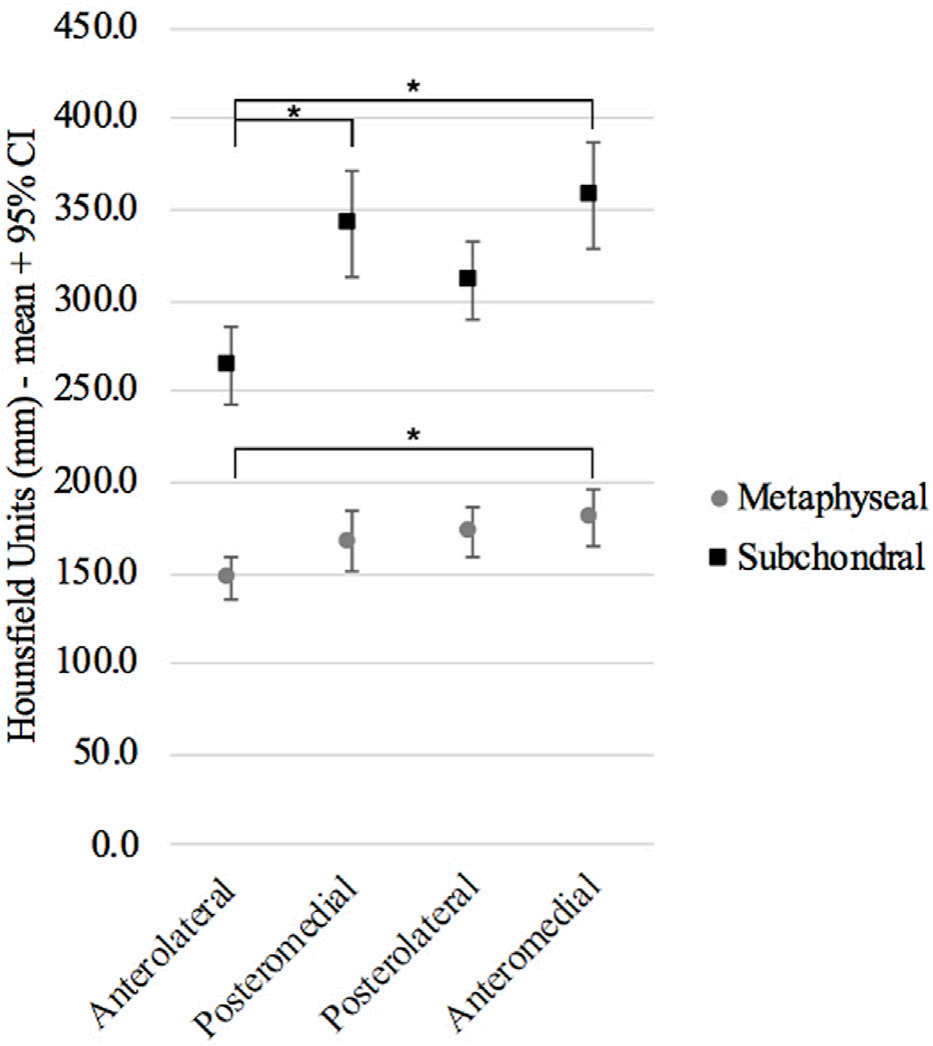
Mean and 95% confidence interval of the 4 regions: anteromedial, anterolateral, posteromedial, and posterolateral in the subchondral and metaphyseal bone.

Additionally, in the comparison of the 4 regions within the subchondral bone we found statistically significant differences (one-way ANOVA for repeated measures F = 34.2, p < 0.001), specifically, higher densities were observed in the anteromedial and posteromedial subchondral regions when compared with anterolateral subchondral region. For the metaphyseal bone, the higher density was observed in the anteromedial compared with the anterolateral (one-way ANOVA for repeated measures F = 16.3, p < 0.001) (Table 1; Figure 3).

The novel method showed an excellent interobserver agreement with an ICC 0.90 (95%CI 0.88–0.93). Age was negatively correlated with subchondral density (Spearman coefficient −0.39, p < 0.01) and metaphyseal density (Spearman coefficient −0.59, p < 0.01). Also, females had a significantly lower total density than males in subchondral bone (303.2 SD:8.4 vs. 347.7 SD 11.2; p = 0.002) and metaphyseal bone (151.1 SD:4.5 vs. 195.8 SD:6.1; p < 0.001)

## Discussion

Our study demonstrates that the tibial plateau is not homogeneous in terms of cancellous bone trabecular density. The subchondral bone exhibits significantly higher density, almost double that of the metaphyseal bone, and medial regions (anteromedial and posteromedial) show higher density than lateral regions in both subchondral and metaphyseal bone.

Although significant differences were found among the distinct regions, the magnitude of these disparities varied considerably. The most pronounced difference was observed between subchondral and metaphyseal bone, with nearly double the HU in the former. Differences among other regions were more subtle. Furthermore, the higher density observed in the medial bone was expected, as the medial plateau bears more load (Fleiss, 2011), and fractures affecting the medial plateau tend to be less comminuted and behave as a larger mass, as described by Schatzker’s classification (Schatzker et al., 1979). It is crucial to determine which of these differences significantly impact screw purchase and should thus be considered distinct areas in FEM analysis of the tibial plateau.

Bones are anisotropic and non-homogeneous structures (Chen et al., 2017). The use of homogeneous isotropic finite elements to describe bone properties has shown promising results in predicting fracture locations and patterns in non-articular regions (Irarrázaval et al., 2021; Mündermann et al., 2008) and studying anatomical variations (Wako et al., 2018; Zagane et al., 2023) and new bone stress distribution (Güvercin et al., 2022). The bone diaphysis derives its main structural properties from the more homogeneous cortical bone, behaving differently from metaphyseal bones, which are significantly influenced by the trabecular bone that dominates this anatomical region. This difference in behavior is one of the features that has led orthopedic surgeons to classify and treat them separately (Moulgada et al., 2023). This distinct behavior is evident in the tibial plateau, where most guidelines support the subchondral location of screws to theoretically increase purchase due to higher density (Müller et al., 1990; Prat-Fabregat and Camacho-Carrasco, 2016). Describing the high density observed in our measurements highlights the relevance of including this feature in future models, differentiating not only cortical and trabecular bone but also subchondral and metaphyseal density regions within the latter.

In FEM studies of proximal tibia fractures, bone properties are often calculated based on Hounsfield units obtained from CT scans (Krause et al., 2018; Chen et al., 2017). In these cases, the density and elastic modulus of the proximal tibia are calculated for each unit in the CT scan, making them impractical for scientific and clinical reproducibility. A simpler approach, defining different stress response limits between subchondral and more distal cancellous bone, could improve the applicability of FEM for patients.

A challenge in measuring density is the lack of standard measurement protocols, likely due to the diverse nature of each bone and its complex geometries. We based our measurements on the surgical approach described by Luo et al. (2010), where observation of fracture patterns has denoted differences between the medial and lateral plateau, as well as the anterior and posterior aspects of each. We developed a novel protocol that was simple enough to allow reproducibility. The excellent interclass correlation found (0.90) allows us to confidently use these measurements to determine the density of each region and predict their mechanical behavior, despite the limitations previously discussed regarding converting HU to density. Additionally, the results of the association of density with age and sex behaved as expected, supporting the measurement technique as capturing an adequate density profile.

The future of FEM should aim to guide surgeons clinically in their decisions. This can only occur if models improve their precision while maintaining an acceptable computational workload. We believe that this simplified region description allows future models to continue the surgical tradition of subchondral hardware placement without overcomplicating the models with element-by-element density determination (Krause et al., 2018; Chen et al., 2017). The main limitations of our study are the limited evidence to safely convert HU to bone density and the lack of a finite element model to highlight the impact of including this subchondral region to determine hardware location. Future studies should focus on the latter to bring us closer to clinical applications of FEM.

## Conclusion

The tibial plateau has a distinct density profile that could explain the lower predictive capability in FEM models that consider this region as homogeneous cancellous bone. And more important, the density profile is related with age and gender, therefore its consideration is critical for an adequate clinical translation of FEM models. Future studies should focus on the implication of these regions on tibial plateau hardware positioning.

## Data Availability

The raw data supporting the conclusions of this article will be made available by the authors, without undue reservation.
